# Non-structural protein 1 of H3N2 influenza A virus induces nucleolar stress via interaction with nucleolin

**DOI:** 10.1038/s41598-017-18087-2

**Published:** 2017-12-19

**Authors:** Yinxia Yan, Yongming Du, Gefei Wang, Kangsheng Li

**Affiliations:** Key Laboratory of Infectious Diseases and Molecular Immunopathology of Guangdong Province, Department of Microbiology and Immunology, Shantou University Medical College, Shantou, Guangdong Province China

## Abstract

The nucleolus is a stress sensor associated with cell cycle progression and a central hub for the replication of pathogenic RNA viruses. However, the role of nucleolus in influenza A virus infection has not been well studied. Here we show that the interaction between NS1 protein of influenza A/Shantou/602/06 (H3N2) and nucleolin, a ubiquitous protein of nucleolus repressed RNA Pol I-dependent transcription via establishing hyper-methylation in the UCE of rRNA gene promoter. NS1 expressed cells showed significant association of ribosomal proteins with MDM2, and p53 accumulation, suggesting induced nucleolar stress. Disruption of the interaction of NS1 with nucleolin or overexpression of nucleolin in NS1 expressed cells revived RNA Pol I-dependent transcription, indicating nucleolin could be one target for NS1 to repress rRNA synthesis of host cells. Our present study suggests that NS1 protein of H3N2 could induce nucleolar stress based on epigenetic alteration of rRNA gene promoter via interaction with nucleolin.

## Introduction

Influenza A virus (IAV) including several subtypes caused severe threat to public health worldwide^1^. Multiple viral proteins of IAV are involved in its infection and pathogenesis^2,3^. Among of these viral proteins, the non-structural protein 1 (NS1) of IAV, which has been identified as a multifunctional viral protein plays roles in disease pathogenesis by modulating a number of cellular immune responses in the infected cells^4^. The N-terminal RNA-binding domain (RBD) and C-terminal effector domain (ED) play essential roles for NS1 protein in antiviral processes^5–8^. NS1 interacts with multiple cellular proteins playing essential roles in immune functions, such as RIG-I, protein kinase R (PKR), cleavage and polyadenylation specificity factor 30 (CPSF30), poly(A)-binding protein II (PABII) and 2′–5′-oligoadenylate synthetase (OAS) to counteract immune responses of the infected hosts^9–13^. It was also reported that NS1 of IAV can induce apoptosis of host cells during infection which served as an important mechanism in viral pathogenesis^14,15^. Cellular localization of NS1 is strain-specific and regulated at least by one or two nuclear localization signals (NLSs), with an N-terminal NLS for all types of NS1 and one additional NLS2 (also annotated as NLoS) for H3N2 NS1. The NS1 protein of H3N2 IAV characteristically distributes inside the nucleolus, and interacts with nucleolin(NCL), suggesting its unique pathogenesis^16–18^.

Nucleolar protein NCL is a multifunctional protein involved in various cellular functions, such as chromatin remodeling, DNA replication, transcription by RNA Pol I or RNA Pol II, rRNA processing and ribosome assembly, cell proliferation and apoptosis^19,20^. NCL interacts with protein and DNA/RNA via its three functional domains, N-terminal acidic-rich domain, central RNA binding domain and C-terminal arginine/glycine rich domain (RGG/GAR domain). It is found that NCL manipulates transcription and stability of mRNA as well as rRNA which is linked to cellular processes influencing cell proliferation and apoptosis involved in pathological events^21,22^. NCL plays important roles in the activation of rRNA transcription and processing as suggested by previous studies^23,24^. And epigenetic regulation of rRNA gene region is proposed to be involved in the rRNA transcription activation^25,26^. Interaction of toxic RNA transcript with NCL is reported to induced nucleolar stress by triggering hyper-methylation of rRNA promoter region to repress rRNA transcription^27^.

Nucleolus is initially regarded as a factory of ribosome production in which ribosomal RNA was synthesized and ribosome was assembled. However, multiple physiological functions such as cell cycle regulation, controlling of aging, small nuclear RNP regulation and coordination of cellular stress responses can also be involved in^28^. Nucleolus is a dynamic nuclear structure assembled in G1 phase and disassembled in M phase and can be involved in stress response and cell cycle arrest^29^. Nucleolus plays roles in sensing stress and could be involved in the regulation of cellular responses. Under stress conditions such as DNA damage, heat shock, nutrient depletion and viral infection, nucleolus reorganization and transmission of stress signal via translocated nucleolar proteins can be triggered^30^. MDM2 mediated stabilization of p53 under nucleolar stress has been well defined even p53 independent pathway was also reported^31^. Ribosomal proteins-MDM2-p53 pathway is a well-defined model that interpreting how translocated ribosomal proteins under nucleolar stress activate p53 dependent cell cycle arrest and apoptosis^32^. Viral infection which was regarded as an important nucleolar stress inducement has been proposed to hijack cellular proteins including nucleolar proteins to facilitating its replication and repress anti-viral responses. NCL is one of these proteins which can be a target for viral proteins during infection^33^.

Multiple IAV viral proteins interact with NCL during infection^34–36^. However, it is currently unknown about the physiological consequences of interaction between IAV NS1 and NCL. In this study, we provided evidence that NS1 derived from influenza A/Shantou/602/06 (H3N2) induces nucleolar stress in A549 cells. We demonstrated that interaction of NS1 and NCL deprives NCL of binding onto ribosomal RNA (rRNA) promoter, which then represses rRNA transcription. The depletion of rRNA triggered nucleolar stress, resulting in p53 dependent apoptosis.

## Results

### Influenza A/Shantou/602/06 (H3N2) induces nucleolar stress in infected A549 cells

**Figure 1:**
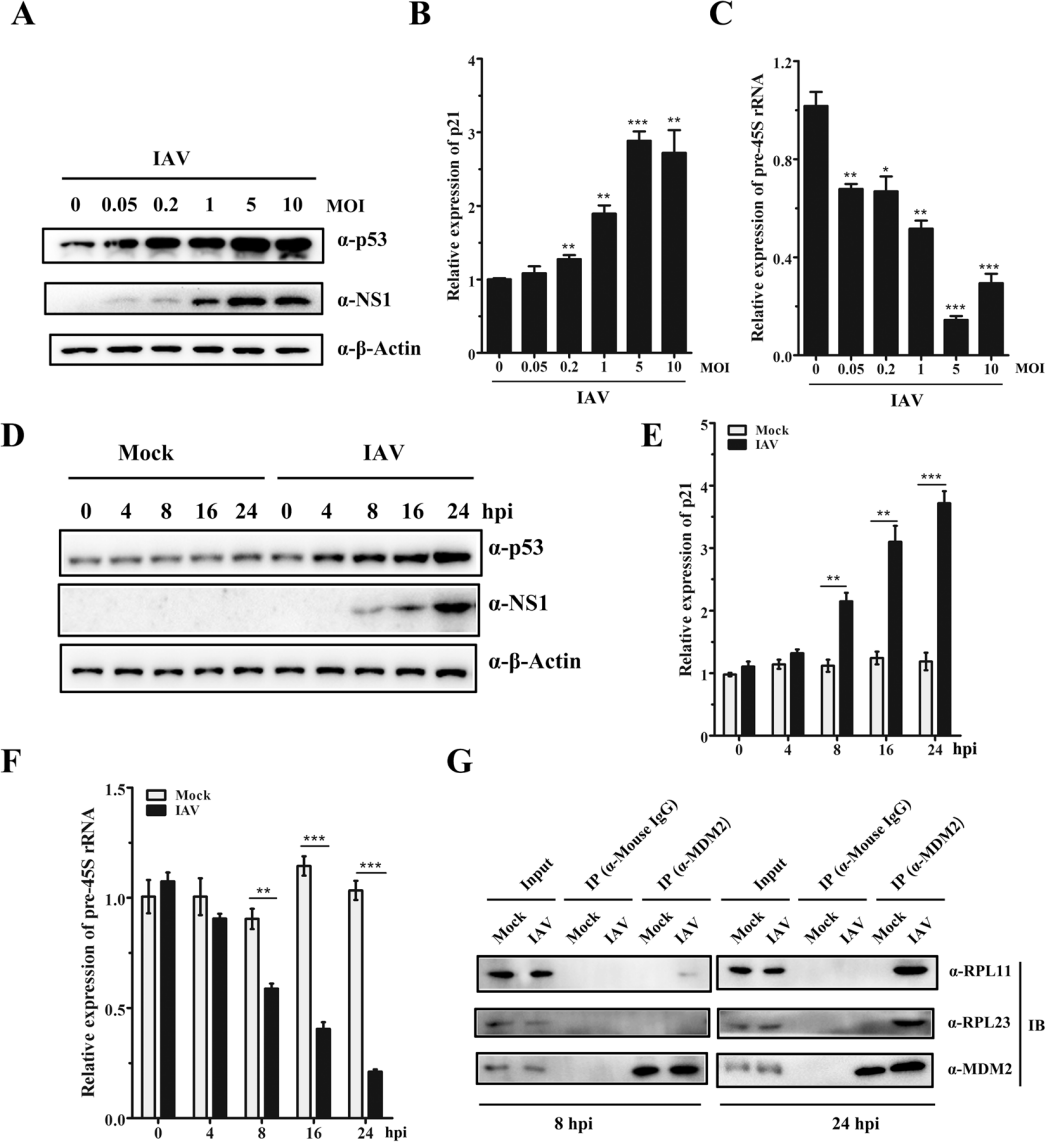
Influenza A/Shantou/602/06 (H3N2) induces nucleolar stress in infected A549 cells. (**A**–**C**) A549 cells grown in 6-well plates were infected with influenza A virus (H3N2) at different MOI ratios (MOI = 0, 0.05, 0.2, 1, 5 and 10) for 24 h. Total proteins were extracted from infected A549 cells and subjected to western blotting to analysis the expression of p53 and viral NS1 protein (**A**). Total RNA was extracted from infected A549 cells and subjected to RT-qPCR to analysis the mRNA level of p21 (**B**) and pre-45S rRNA (**C**). (**D**–**F**) A549 cells grown in 6-well plates were mock-infected (mock) or infected with influenza A virus (H3N2) (IAV) at MOI 5. Cells were harvested at different time points post-infection (0, 4, 8, 16 and 24 h). Western blot analysis of p53 and viral NS1 rotein (**D**). RT-qPCR of p21 and pre-45S rRNA (**E** and **F**). Results show the mean of the three experiments ± SD and are normalized to the β-actin gene. *p < 0.05, **p < 0.01, ***p < 0.001 (*t-test*). (**G**) Co-immunoprecipitation of RPL11 and RPL23 with MDM2 in mock-infected or infected with influenza A virus cells at 8 hpi or 24 hpi. For each, 5% cell lysate was served as input.

It has been previously demonstrated that RNA viruses interact with nucleolus to induce nucleolar stress response of host-cell and recruit nucleolar proteins to facilitate virus replication^33^. NS1 protein derived from H3N2 influenza A virus has been shown to interact with functional nucleolar protein, nucleolin and fibrillarin^18^. We therefore considered the possibility that H3N2 IAV possibly modulates nucleolar function in infected cells. A549 cells were infected with influenza A/Shantou/602/06 (H3N2) virus at different MOI ratios (MOI = 0, 0.05, 0.2, 1, 5 and 10) for 24 h. Both of the level of pre-45S rRNA and the mRNA level of p21 were measured by RT-qPCR and intracellular p53 was monitored by western blot. Viral NS1 protein was used as an indicator of virus replication. Protein level of p53 (Fig. 1A) and mRNA level of p21 (Fig. 1B) in IAV-infected cells were elevated in an MOI-dependent manner, compared with that in mock-infected cells. pre-45S rRNA levels in infected cells were decreased dramatically with the increase of MOI (Fig. 1C). Next, we assessed the levels of p53, p21 and pre-45S rRNA in IAV-infected A549 cells at MOI 5 at various time points post-infection. Mock-infected cells revealed a relatively constant level of p53 protein, p21 mRNA and pre-45S rRNA whereas in the parallel cultures of IAV-infected cells, p53 protein and p21 mRNA levels increased, pre-45S rRNA levels declined in a time-dependent manner (Fig. 1D–F).

Human RPL11 and RPL23 have been reported to be involved in nucleolar stress by binding and sequestering the MDM2 to ensure p53 stabilization and activity^37,38^. We next investigated the interaction between RPLs (RPL11 and RPL23) and MDM2 during different time points post-infection (8 hpi and 24 hpi). As shown in Fig. 1G, we observed a weak binding between RPL11 and MDM2 at 8 hpi and a strong interaction at 24 hpi in IAV-infected cells (Fig. 1G, upper panel). We also detected an obvious interaction between RPL23 and MDM2 at 24 hpi in IAV-infected cells, though failed to detect the interaction at 8 hpi (Fig. 1G, lower panel). Collectively, all these data indicated that influenza A/Shantou/602/06 (H3N2) induces nucleolar stress in infected A549 cells.

### NS1 of influenza A/Shantou/602/06 (H3N2) induces nucleolar stress in host cells

**Figure 2:**
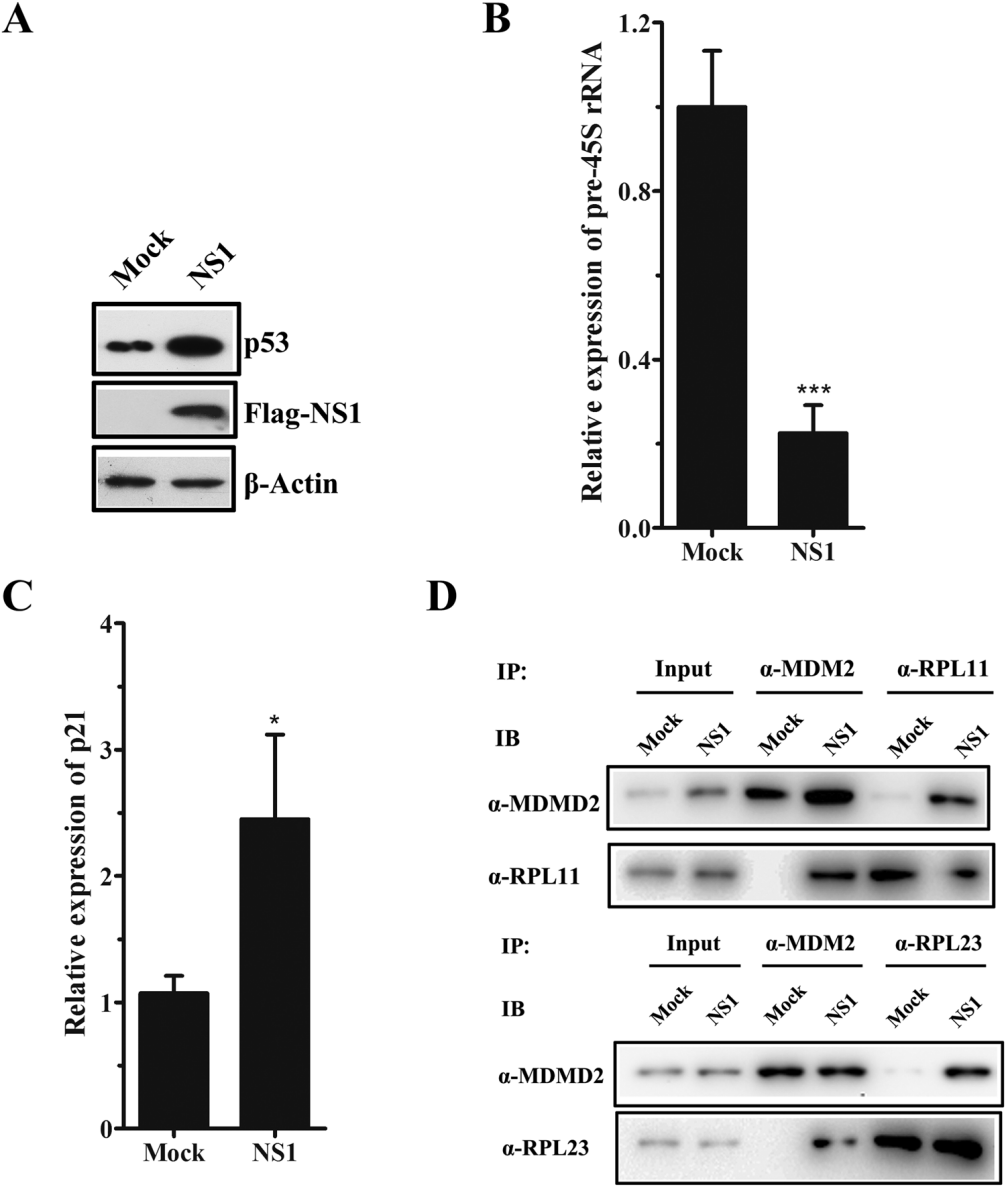
NS1 of influenza A/Shantou/602/06 (H3N2) induces nucleolar stress in host cells. A549 cells grown in 6-well plates were mock-transfected or transfected with NS1 for 36 h. (**A**) Cell lysates were immunoblotted with anti-p53 antibody and anti-Flag antibody. β-Actin was served as a loading control. RT-qPCR analysis of pre-45S rRNA (**B**) and p21 (**C**) in mock-transfected or NS1-transfected A549 cells. Results show the mean of the three experiments ± SD and are normalized to the β-actin gene. *p < 0.05, ***p < 0.001 (*t-test*). (**D**) Co-immunoprecipitation of RPL11 (upper panel) and RPL23 (lower panel) with MDM2 in mock-transfected or NS1-transfected A549 cells. For each, 5% cell lysate was applied as input.

Previous studies reported that NS1 protein of influenza A/Udorn/72 (H3N2) virus specifically localized in nucleolus and interacted with nucleolar proteins, nucleolin and fibrillarin via its C-terminal NLS^18^. Even several amino acids in NLSs of A/Shantou/602/06 NS1 differ from that of A/Udorn/72 NS1(Figure S1), NS1 of influenza A/Shantou/602/06 (H3N2) showed similar co-localization and interaction with NCL as NS1 of A/Udorn/72 (H3N2) virus (Figure S2). To determine whether the nucleolar stress induced by the A/Shantou/602/06 (H3N2) virus correlated with its NS1 protein, A549 cells were transiently transfected with empty vector (mock) or Flag-tagged NS1 plasmid (NS1) for 36 h. p53 accumulation was detected in NS1-transfected cells (Fig. 2A), and pre-45S rRNA level was significantly decreased (Fig. 2B), compared with the mock, suggesting that nucleolar stress was induced. In addition, p21 mRNA level in NS1-transfected A549 cells was increased by about 2.5-fold compared with the mock (Fig. 2C). Besides, interaction of MDM2 with RPL11 or RPL23 was also identified in NS1-transfected cells by co-immunoprecipitation (Fig. 2D). These results indicate that NS1 protein of A/Shantou/602/06 (H3N2) could induce nucleolar stress in A549 cells.

### NS1 induces nucleolar stress dependent on its interaction with NCL

**Figure 3:**
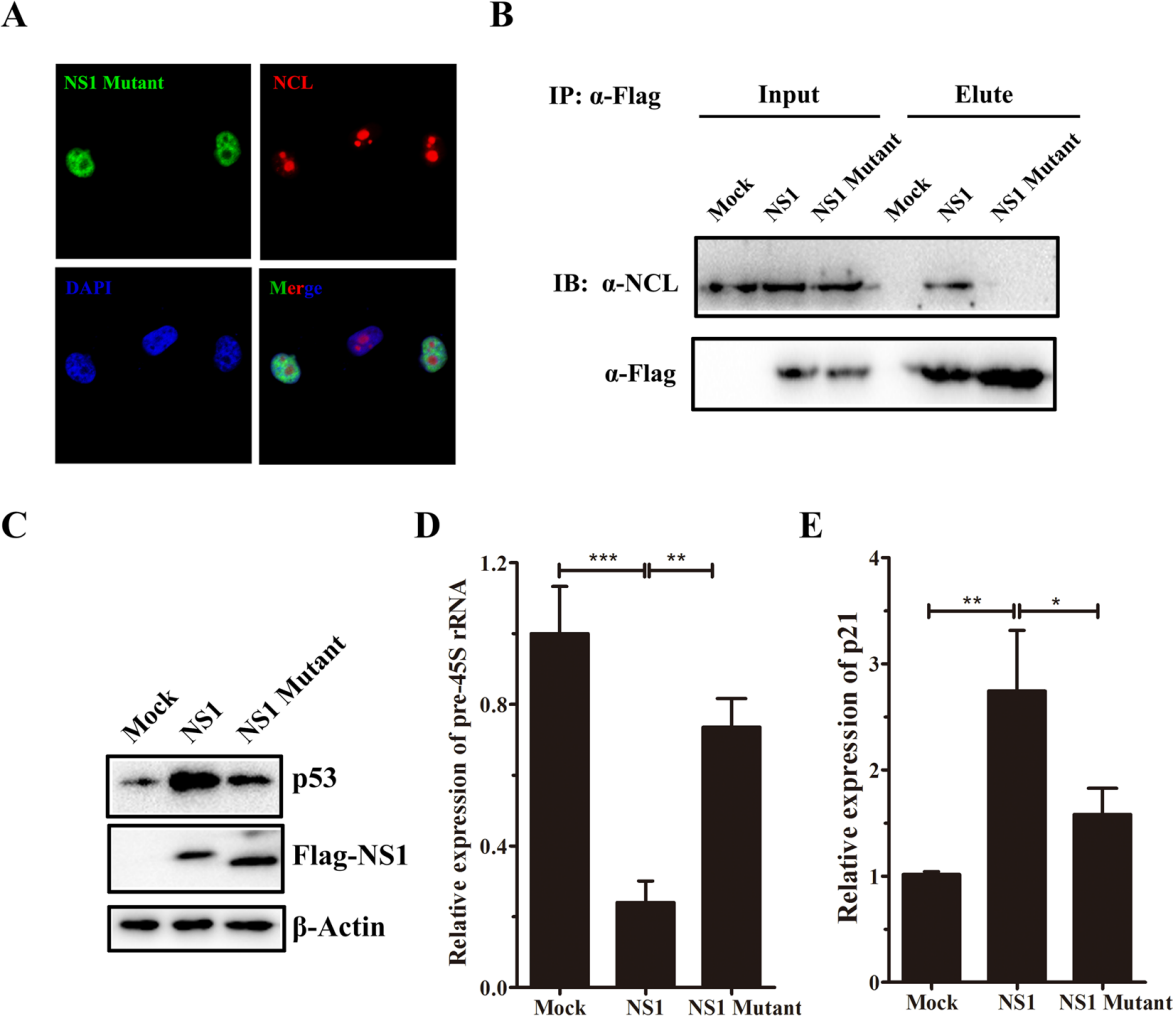
NS1 of Influenza A/Shantou/602/06 (H3N2) induces nucleolar stress depending on its association with NCL. (**A**) Immunofluorescence staining of NS1 Mutant (218–230 aa truncated mutant) (Green), or NCL (Red) in A549 cells. (**B**) Lysates from mock-transfected, NS1-transfected or NS1 Mutant-transfected A549 cells were subjected to immunoprecipitation using anti-Flag antibody and immunoblotted with anti-NCL or anti-Flag antibodies. 5% cell lysate was served as input. (**C**) Cell lysates harvested 36 h post-transfection were processed for western blot with p53 antibody. β-actin was used as the loading control. (**D**) RT-qPCR analysis of pre-45S rRNA and p21 (E) in transfected A549 cells. Results show the mean of the three experiments ± SD and are normalized to the β-actin gene. *p < 0.05, **p < 0.01, ***p < 0.001 (*t-test*).

To figure out whether NS1 induce nucleolar stress dependent on its interaction with NCL, we generated truncation of NS1 (NS1 Mutant) to abolish the interaction of NS1 with NCL (Figure S1). It has been reported that NS1 protein of influenza A H3N2 with H3K4-like motif “ARSK” at its carboxyl terminus could bind to host cellular proteins such as PAF1 complex, CHD1 and WDR5. This histone mimicry facilitates the virus to hijack host proteins and modulate inducible antiviral gene expression^39,40^. Melen *et al*. proposed an NoLS sequence at the C-terminal of Udorn72 (H3N2) NS1 protein is important for nucleolar localization and NCL interaction, inside which critical amino acids 219, 220, 224, 227, 229, 231, and 232 are involved in targeting the NS1 protein into the nucleus and nucleolus^18^. The “ARSK” motif is also inside this region which might also be involved, in light of NCL has histone chaperon activity. In our study, we generated truncation of A/Shantou/602/06 (H3N2) NS1 lack of 218–230 amino acids which sufficiently alternated nucleolar localization and NCL interaction activity. The truncation comprised nucleolar accumulation of NS1 suggested by the result of immunofluorescence microscopy (Fig. 3A), and efficiently abolished its binding with NCL suggested by the result of co-immunoprecipitation (Fig. 3B). We next compared nucleolar stress induction effect between full-length NS1 (NS1) and truncated NS1 (NS1 Mutant). RT-qPCR analysis of pre-45S rRNA showed rRNA transcription in NS1-transfected cells was dramatically repressed, whereas no significant decrease of rRNA level in NS1 Mutant-transfected cells was detected (Fig. 3D). The ability of inducing p53 protein accumulation (Fig. 3C) and p21 up-regulation (Fig. 3E) of NS1 was also comprised for the truncated mutant (Fig. 3C), suggesting that the induction of nucleolar stress can be depended on the interaction between NS1 and NCL.

### NCL is a target protein for NS1 to repress rRNA synthesis and induce nucleolar stress

**Figure 4:**
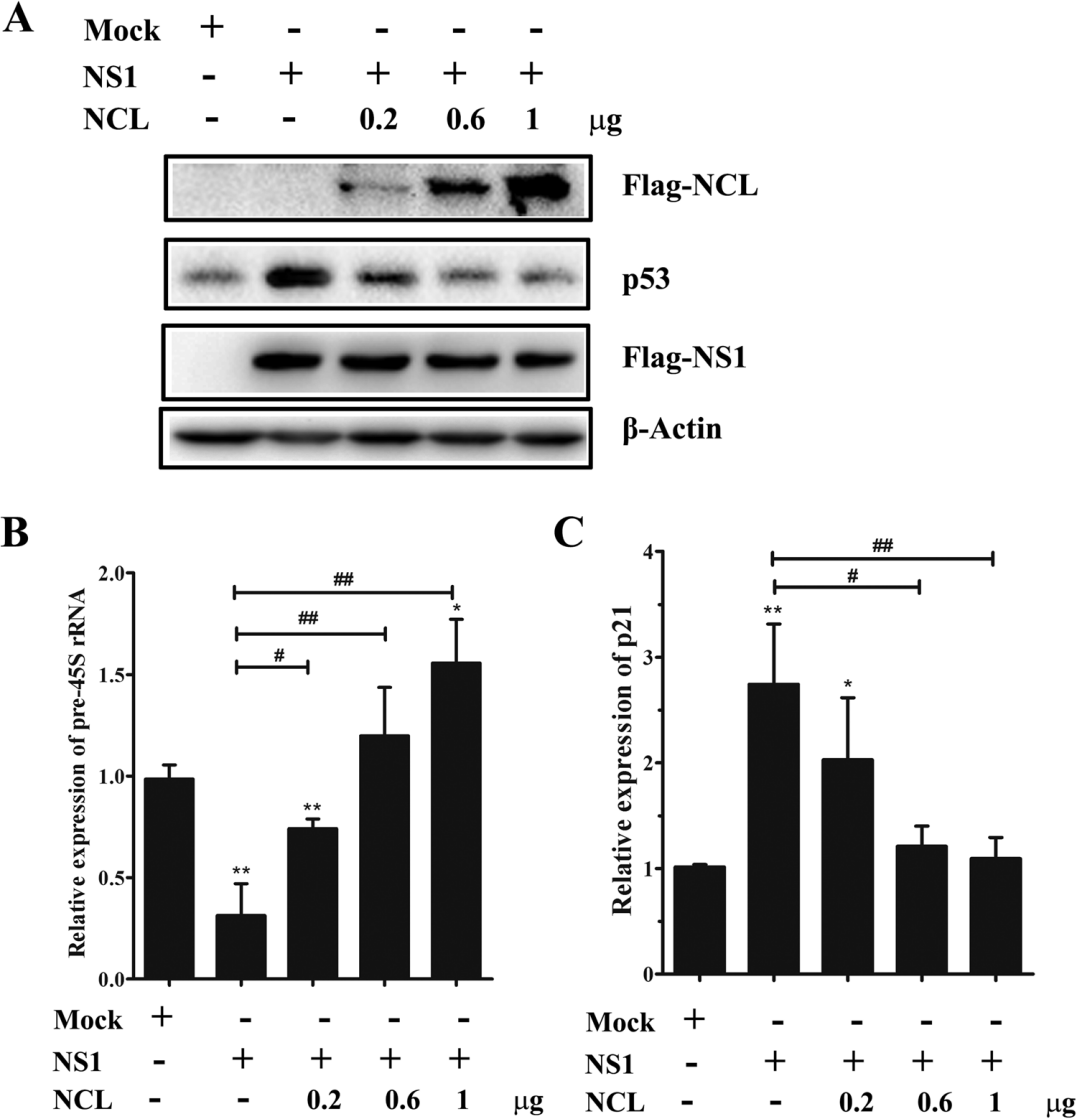
Dose-dependent effect of NCL overexpression on H3N2/NS1-induced nucleolar stress. Western blot analysis was used to confirm 3 x Flag-tagged NCL overexpression and its effect on p53 expression level in NS1-transfected A549 cells. β-actin was used as a loading control (**A**). Total RNA extracted from different amount of NCL constructs and NS1 co-transfected A549 cells and subjected to RT-qPCR to measure the mRNA level of pre-45S rRNA (**B**) and p21 (**C**). “−” Indicates cells without NCL overexpression. Error bars represent ± SD from three independent experiments. *p < 0.05, **p < 0.01, ^#^ *p* < 0.05, ^##^ *p* < 0.01 (*t-test*).

It has been previous reported that NCL knockdown represses rRNA transcription and induces nucleolar stress in HEK293T cells^27^. Herein, we conducted siRNA experiments targeting NCL in A549 cells, and obtained a similar effect on pre-45S rRNA transcription, p53 accumulation and p21 up-regulation (Figure S3). We next determined whether overexpression of NCL could rescue NS1-induced dysregulation of rRNA transcription. Our results showed that NCL overexpression rescued rRNA transcription in NS1-transfected A549 cells in a dose-dependent manner (Fig. 4B). We further observed that NCL overexpression suppressed the NS1-induced p53 accumulation and p21 up-regulation (Fig. 4A and C). These results indicate that NS1 induced nucleolar stress triggered by rRNA transcription repression can be reversed by NCL overexpression, suggesting that NCL can be served as a target protein for NS1 and should be involved in NS1 induced nucleolar stress.

### NS1 triggered hyper-methylated state of rRNA promoter region and blocked RNA Pol I recruitment

**Figure 5:**
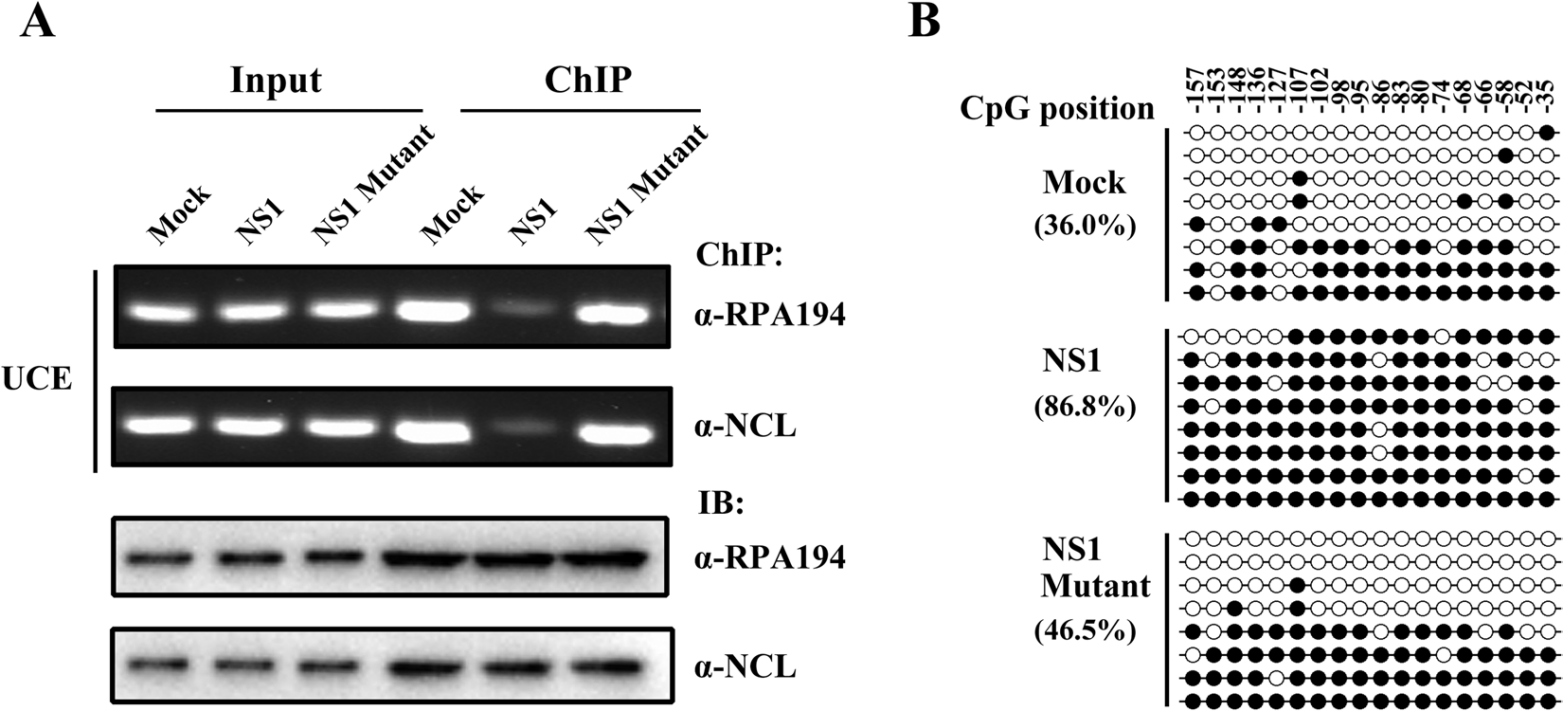
NS1 alternates methylation state of rRNA promoter region and blocks RNA pol I recruitment. (**A**) Chromatin immunoprecipitation of the largest subunit of RNA polymerase I (RPA194) or NCL with UCE in mock-transfected, NS1-transfected or NS1 mutant-transfected A549 cells. Immunoprecipitated DNA was analyzed by PCR using the primers shown in Table S3. (**B**) Bisulfate sequencing analysis of CpG methylation state of rRNA gene UCE region in mock-transfected, NS1-transfected or NS1 Mutant-transfected A549 cells. Closed and open circles donate methylated and unmethylated CpG sites. Each row represents the sequence of a clone, and each column represents the position of CpG sites in human UCE. Precentages represent CpG methylated ratios. Displayed sequences of every 8 clones were randomly selected from the total 24 clones of three independent experiments.

The mechanism underneath the nucleolar stress induced by NS1 should involve rRNA transcription. Previous studies suggested epigenetic regulation of rRNA gene by NCL could play roles in activation or repression of rRNA transcription^25–27^. We first tested whether NS1 could affect association of NCL or RNA Pol I with rRNA gene promoter UCE region by ChIP assay. The result showed dramatically reduced association of NCL or RPA194 with UCE of rRNA gene promoter in wild type but not truncated mutant NS1 expressed A549 cells (Fig. 5A). We also detected the similar effect in A/Shantou/602/06 (H3N2) virus infected A549 cells (Figure S4). The result indicated NS1 modulated the association of NCL or RPA194 with UCE. Hyper-methylation on UCE of rRNA gene promoter has been reported to be contributed to rRNA transcription inbibition^27^. We next measured the methylation state of CpG sites within UCE of rRNA gene promoter in mock-transfected, NS1-transfected and NS1 Mutant-transfected A549 cells, and found that NS1 expression significantly triggered the hyper-methylation of UCE but NS1 mutant without NCL binding capacity showed very slight change of methylation level on UCE (Fig. 5B). The result suggested interaction between NS1 and NCL contributed to hyper-methylation of UCE and thereby compromised RNA Pol I recruitment which determined rRNA transcription efficiency.

## Discussion

NS1 of IAV is a multi-functional protein and an important virulence factor that plays pivotal roles in virus infection via its interaction with multiple cellular factors. The intercellular localization of NS1 has impact on its function. For example, NS1 in the cytoplasm has been reported to bind to PKR, inhibiting the activation of PKR and facilitating the viral protein synthesis^10^, whereas, NS1 in the nucleus inhibits its post-transcriptional processing of pre-mRNA by binding to CPSF30 and PABII^11,12^. The present study indicated that NS1 of H3N2 subtype IAV targeted to the nucleolus and induced nucleolar stress via its interaction with NCL.

Nucleolus is a gateway for viral infection and a hub for cellular stress^33^. Nucleolar stress can be induced by multiple exogenous stimuli such as exposure of irradiation, nucleotide depletion, heat shock and viral infections. Inhibition of rRNA synthesis is a major mechanism of nucleolar stress induction^30^. In the present study, we report that influenza A/Shantou/602/06 (H3N2) virus infection represses pre-rRNA transcription, stabilizes p53, up-regulates p21 and induces MDM2 binding to ribosomal proteins, indicating nucleolar stress can be triggered in infected A549 cells. We propose that NS1 protein of A/Shantou/602/06 (H3N2) should be, at least in part, responsible for the nucleolar stress response based on our experimental data. In NS1-transfected A549 cells, dramatic repression of pre-rRNA transcription, significant p53 accumulation and strong binding of ribosomal proteins with MDM2 suggested nucleolar stress induced by NS1. Further study demonstrate that interaction of NS1 with NCL is responsible for depletion of pre-45S rRNA transcription in human cells and nucleolar stress. Our results suggeste that interaction between NS1 and NCL or depletion of endogenous NCL leaded to repression of pre-45S rRNA transcription and nucleolar stress, which can be revived by exogenously expressed NCL in dose dependent manner. An important pathway for nucleolar stress induced apoptosis is the proposed ribosomal protein-MDM2-p53 pathway. Ribosomal proteins under nucleolar stress translocate and directly bind to MDM2, inhibiting its activity towards p53^32^. We also evaluated apoptosis induction of NS1 proteins derived from three different IAV strains by measuring caspase 3/7 activity, and found that H3N2/NS1, similar to H5N1/NS1 and H7N9/NS1^14,15^, could activate caspase 3/7 significantly, indicating that nucleolar stress trigged by NS1-induced rRNA depletion might play a role in cell apoptosis (Figure S5). Taken together, our study indicates nucleolar multiple-functional protein NCL as a targeting protein which can be bound by IAV viral protein NS1, resulting in nucleolar stress in host cells.

NCL regulates rRNA biogenesis and ribosome assembly as indicated by accumulating studies^23–27^. The mechanism of how NCL regulates rRNA biogenesis is not defined, but several studies indicated that epigenetic modulation of rRNA gene at DNA or chromatin level might be involved in. NCL was first described as a negative regulator of rRNA transcription since microinjection of NCL in Xenopus oocytes reduced pre-rRNA^41^. However, later studies proposed NCL as a positive regulator of rRNA transcription^23,25–27^. Accumulating evidences obtained from either knock down of NCL via siRNA or conditional knockout suggested that depletion of NCL repressed rRNA transcription^42^. Rickards *et al*. demonstrated that NCL facilitated RNA polymerase I transcription of chromatin templates *in vitro* ^25^. In our study, we detected compromised rRNA transcription in NCL depleted cells or NS1 expressed cells, and both of which can be recovered rRNA transcription by overexpression of NCL.

Epigenetic regulations involved both chromatin state and DNA methylation state of rRNA gene region could contribute to the rRNA transcription regulation. NCL counteracts with macro H2A1, prevent from heterochromatin establishment at rDNA^43^. Cong and colleagues found that depletion of NCL resulted in an increased heterochromatin mark H3K9me2 and a decreased H4K12Ac and H3K4me3 euchromatin histone marks in rRNA genes. Their ChIP- seq experiments showed an enrichment of NCL in the ribosomal DNA (rDNA) coding and promoter regions. Their data indicated NCL was preferentially associated with unmethylated rRNA genes and its depletion resulted in the accumulation of RNA Pol I at the beginning of the transcription unit and a decreased occupancy of UBF along the coding and promoter regions^23^. Tsoi *et al*. demonstrated that expanded CAG RNAs inhibited RNA Pol I dependent rRNA transcription via interact with NCL. And the hyper-methylation of UCE resulted from interaction between expanded RNAs and NCL should be responsible for the inhibition of rRNA transcription^27^. Our results suggested the depletion of NCL or interaction between NS1 and NCL induced a hyper-methylation state of rRNA gene at UCE region and compromised recruitment of RNA Pol I, which could be responsible for repressed rRNA transcription.

NCL is one of the most abundant nucleolar non-ribosomal proteins and has been shown to be a target protein for multiple human viruses during infection^44^. It has been reported that viral proteins interact with NCL and translocate NCL out of nucleolus or nucleus. IAV viral proteins, such as hemagglutinin (HA), nucleoprotein (NP), and NS1 have been reported to interact with NCL during infection. HA interacted with cell surface NCL which is required for virus internalization^34^. NP was demonstrated as an interaction partner of endogenous NCL during early infection in cytoplasm after its translocation^35,36^. Terrier *et al*. studied the NCL interaction with H3N2 IAV NS1 and NP proteins during infection and observed interaction between NS1 and NCL in early stage of infection. NCL was translocated into cytoplasm in late stage of infection and interacts with NP. NS1 seems to interact with nucleolar localized NCL during early IAV infection, and translocated after nucleolar architecture reorgnized^36^. In our study, NS1 protein of H3N2 IAV was expressed in A549 cells to focus on the consequence of NS1-NCL interaction. Our results suggest that inhibition of rRNA gene transcription by NS1 resulted in induction of nucleolar stress. The compromised rRNA transcription could be related with epigenetic modulation of rRNA gene promoter. Interaction between NS1 and NCL should be responsible for such epigenetic modulation. It is not currently known how NCL maintain the DNA methylation state of rRNA gene. The mechanism may vary among different study models.

Fibrillarin, another nucleolar protein, which plays important roles in rRNA processing, rRNA methylation and ribosome assembly has also been reported as an interaction partner of H3N2 NS1^18,45,46^. In light of the important functions of fibrillarin in rRNA post transcription modification and its physical interaction with NS1 of H3N2 IAV, it is also a potential target protein of nucleolar localized NS1 in infected cells and might be involved in nucleolar stress induction. Thus, NCL might not be the sorely nucleolar target of NS1 in the infection process of H3N2 IAV. Further study will benefit on the understanding of comprehensive H3N2 NS1-nucleolus interactive network that determine the viral infection process and host cellular functions.

**Figure 6:**
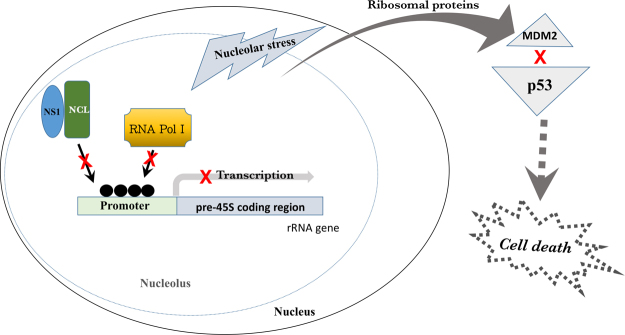
Proposed model of NS1-induced nucleolar stress. Interaction of H3N2/NS1 with NCL compromises NCL association with rRNA gene and results in hyper-methylation state of UCE region of rRNA gene promoter. Hyper-methylated UCE leads to reduced recruitment of RNA Pol I to rRNA gene promoter and inhibition of rRNA transcription, which represses rRNA synthesis, and thereby induces nucleolar stress. Under this rRNA shortage circumstance, free ribosomal proteins interact with MDM2 E3 ubiquitin ligase. The interaction abolishes the negative regulation capacity of MDM2 towards p53 and leads to p53 accumulation, then triggers cell apoptosis.

The present study shows NS1 derived from A/Shantou/602/06 (H3N2) induces nucleolar stress via its interaction with NCL. Herein, we proposed a model for interpreting the mechanism of NS1 induced nucleolar stress based on our experimental data (Fig. 6). NS1 of IAV interacts with NCL inhibiting its association with rRNA UCE region, which results in hyper-methylation of UCE. The recruitment of RNA Pol I could be compromised under hyper-methylated UCE condition, leading to repression of rRNA transcription and activation of nucleolar stress pathway. Depletion of rRNA promotes the interaction between free ribosomal proteins and MDM2, inhibiting p53 degradation. The accumulated p53 may trigger apoptosis of infected cells. Wang *et al*. reported that Mdm2-mediated ubiquitination and stabilization of p53 should be involved during influenza infection^47^. NS1 was also reported to interact with and stabilize p53, resulting in an alteration of p53-responsive genes expression in a promoter-dependent manner^48^. These studies suggest NS1 also regulates p53 directly. Apoptosis which was regarded as anti-viral response can be taken advantage by IAV virus to facilitate its replication^49^. In our study, we proposed a model of nucleolar stress mediated apoptosis. Multiple nucleolar proteins can be hijacked by viral proteins under nucleolar stress, which facilitated virus replication^33^. Under nucleolar stress condition, nucleolar proteins translocated from nucleolus to cytoplasmic which can be taken advantage by virus^30^. Terrier *et al*. reported that NCL interacted with NS1 of H3N2 IAV at early phase and interacted with NP at late phase of infection. The modification of nucleolus architecture was detected. NP co-translocation with NCL was proposed to facilitate viral RNP trafficking and viral replication^36^. We assume that interaction between NS1 and NCL in early infection might contribute to nucleolus structural modification under its induced nucleolar stress followed by NP-NCL co-translocation.

In conclusion, our present study suggests that NS1 protein of H3N2 could induce nucleolar stress based on epigenetic alteration of rRNA gene promoter via interaction with NCL, which might lead to p53 dependent apoptosis

## Materials and Methods

### Viruses and cells

Viral stocks of influenza A/Shantou/602/06 (H3N2) were prepared in MDCK cells and virus titer was determined by plaque assay. Virus stocks were diluted with serum-free medium for adsorption. A549 and MDCK cells were cultured in DMEM medium supplemented with 10% FBS and 1% penicillin-streptomycin at 37 °C with 5% CO_2_. For viral infection, A549 cells were absorbed with influenza A virus at the multiplicity of infection (MOI) of 0.05, 0.2, 1, 5 or 10 for 1 h at 37 °C, washed with PBS, and incubated in the DMEM containing 1% FBS and 1 μg/ml TPCK trypsin (Sigma) at 37 °C. For mock infection, the procedure was performed in an identical fashion to the viral infection using PBS as the inoculum. All the experiments involved biosafety were conducted in BSL-2 level bio-containment.

### Antibodies

Antibodies used in this study were listed as follows: anti-p53 monoclonal antibody (1C12, Cell Signaling Technology), anti-p53 monoclonal antibody (7F5, Cell Signaling Technology), anti-Flag monoclonal antibody (M2, Sigma), anti-NS1 monoclonal antibody (NS1-23-1, Santa Cruz), anti-MDM2 polyclonal antibody (C18, Santa Cruz), anti-RPL11 polyclonal antibody (SAB2700779, Sigma), anti-RPL23 polyclonal antibody (I-20, Santa Cruz), anti-nucleolin monoclonal antibody (C23, Santa Cruz), anti-nucleolin polyclonal antibody (N2662, Sigma), anti-RPA194 monoclonal antibody (C-1, Santa Cruz), anti-β-actin monoclonal antibody (AC-15, Sigma), a horseradish peroxidase (HRP) conjugated goat anti mouse IgG antibody (Sigma), goat anti-rabbit IgG antibody (Sigma).

### RNA preparation, RT-PCR and Real-time Quantitative PCR (qPCR)

Total RNA was extracted from cells using TRIzol reagent (Invitrogen) while viral RNA was purified using the NucleoSpin® RNA Virus kit (Macherey-Nagel). Reverse transcription was conducted using the PrimeScript® RT reagent Kit (Takara) following the manufacturer’s manual. Real time qPCR was carried out in an Eco Real-Time PCR System (Illumina, USA) using ChamQ^TM^ SYBR® qPCR Master Mix (Vazyme, China). All the transcription specific primers and qPCR primers used are listed in Table S1.

### Plasmids and Transfection

Flag-tagged NS1, Flag-tagged NS1 Mutant were conducted by inserting the full-length (1-230 aa) and truncated (1-217 aa) viral NS1 cDNA into pcDNA3-Flag vector (a modified pcDNA3 vector with N-terminal Flag tag). Flag-tagged NCL was conducted by inserting human nucleolin cDNA into p3 x FLAG-CMV-7.1 vector (Sigma). Cloning primers used in this study are listed in Table S2. A549 cells were transfected using Lipofectamine 2000 (Invitrogen) according to the manufacture’s protocol.

### Immunofluorescence

A549 cells were inoculated on glass coverslips and transfected with indicated DNA for 24 h. The transfected cells were fixed with 4% paraformaldehyde in PBS for 5 min and permeabilized with 0.2% Triton X-100, 0.04% SDS in PBS for 5 min. After blocking in 10% normal goat serum (Boster, Wuhan, China) at room temperature for 30 min, cells were incubated overnight at 4 °C with primary antibody against Flag epitope or NCL. The appropriate Alexa-Fluor-coupled secondary antibody (Invitrogen; 1:750) was applied for 1 h at room temperature. The cells were mounted in ProLong Gold antifade reagent with 6-diamidino-2-phenylindole (DAPI) (Life Technologies) and analyzed under an Olympus FV1000 laser confocal microscope (Olympus, Japan).

### Co-immunoprecipitation

For NS1 transfected cells, A549 cells grown in 100 mm plates were transiently transfected with 15 μg of Flag-tagged NS1 (NS1), Flag-tagged NS1 mutant (NS1 Mutant) or empty vector (Mock) and incubated for 36 h. For influenza A virus infection, A549 cells infected with influenza A/Shantou/602/06 at a MOI of 5 and incubated for 8 h or 24 h post-infection, for mock-infection, PBS was used to replace virus particles. Both transfected and infected cells were lysed in a TNE buffer (20 mM Tris-HCl, PH7.4, 150 mM NaCl, 5 mM EDTA, 0.5% Nonidet P-40) with protease inhibitor cocktail (Cell signaling technology), and 5% of each lysate was served as input control. Lysates were precleared by incubation with protein A/G magnetic beads (General Eletric) for 1 h at 4 °C, and were then incubated with anti-Flag, anti-NCL, anti-MDM2, anti-RPL11, anti-RPL23 antibodies on a rotating wheel overnight at 4 °C. Protein A/G magnetic beads were added and mixtures were further incubated for another 2 h at 4 °C. The magnetic beads were washed three times in TNE buffer. The antibody-antigen complexes were eluted in 2 x SDS-PAGE loading buffer by boiling for 3 min. Both the input and elute of samples were separated by SDS-PAGE and analyzed by western blotting with the indicated antibodies.

### Chromatin immunoprecipitation (ChIP) Assay

ChIP was performed with EZ ChIP Kit (Millipore) according to the manufacturer’s protocol. Briefly, 1 x T75 flask of cells were fixed for 10 min with 1% formaldehyde and harvested in 1 ml lysis buffer. The cell lysates were sonicated to yield DNA fragments between 200 and 500 bp. Cell lysates were immunoprecipitated overnight at 4 °C with the following antibodies: anti-RPA194 and anti-NCL. 5% of cell lysate was served as input control. Protein G agarose beads were then added and the mixtures were further incubated on a rotating wheel for 2 h at 4 °C. The agarose beads were washed before eluted following the kit manual. Cross-links were reversed by heating for 6 h at 65 °C. RNA was digested by RNase A for 1 h at 37 °C. The DNA fragments were then purified. The purified DNA fragments were amplified by PCR, using the primers in Table S3.

### Bisulfite Sequencing for DNA Methylation Analysis

Genomic DNA was extracted with a PureLink® Genomic DNA Mini Kit (Life Technology), and the bisulfite conversion reaction was performed using the EpiTect Bisulfite kit (QIAGEN) according to the manufacturer’s instructions. The rRNA promoter spanning −310 to −28 bp was amplified using the primers in Table S4. The amplified products were cloned into pEGM-T Easy Vector (Promega) and sequenced. For each PCR product, eight clones were sequenced to analyze the UCE methylation state in the *rRNA promoter* region.

## Electronic supplementary material

Supplementary Information
