# Supplementary material for: Non-structural protein 1 of H3N2 influenza A virus induces nucleolar stress via interaction with nucleolin
Source: Sci Rep. 2017 Dec 19;7:17761. doi: 10.1038/s41598-017-18087-2 (PMC5736645; doi:10.1038/s41598-017-18087-2)
Supplement: Supplementary file 1 — Supplementary Information [file 41598_2017_18087_MOESM1_ESM.pdf]

Non-structural protein 1 of H3N2 influenza A Virus Induces nucleolar stress via Interaction with Nucleolin

Running Title: NS1-NCL Interaction Induces nucleolar stress

Yinxia Yan, Yongming Du, Gefei Wang* and Kangsheng Li*

Key Laboratory of Infectious Diseases and Molecular Immunopathology of Guangdong Province, Department of Microbiology and Immunology, Shantou University Medical College, Shantou, Guangdong Province, China

*Correspondence to: Gefei Wang, E-mail: geifeiwang@stu.edu.cn and Kangsheng Li, E-mail: ksli@stu.edu.cn; Key Laboratory of Infectious Diseases and Molecular Immunopathology of Guangdong Province, Department of Microbiology and Immunology, Shantou University Medical College, 22 Xinling Road, Shantou Guangdong, 515041, China. Fax: +86 754 8890417. Tel: +86 754 88900456.

Supplemental Figures

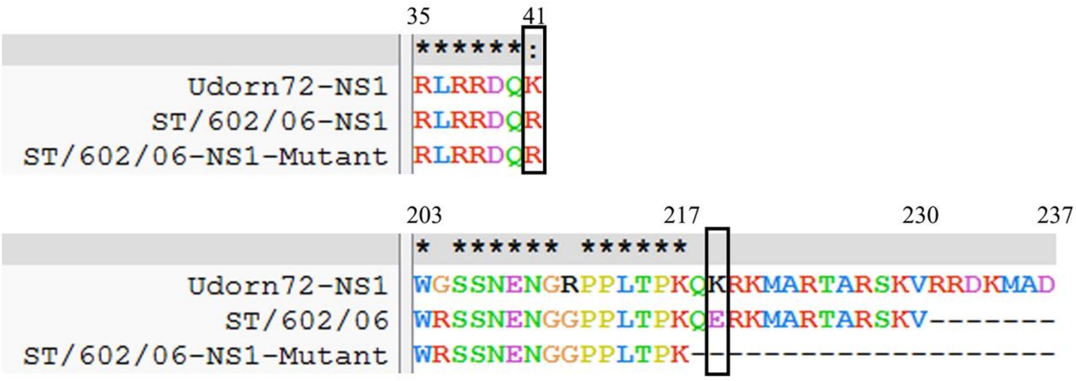

Figure S1. Alignment of NLSs between NS1 proteins from A/Shantou/602/2006 (H3N2) and A/Udorn/1972 (H3N2). NLSs of NS1 Mutant (1-217aa) used in this study was also presented.

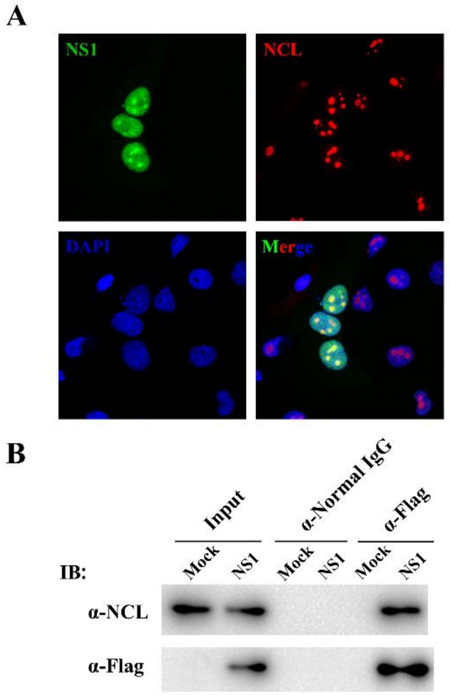

Figure S2. NS1 of A/Shantou/602/2006 (H3N2) co-localizes and interacts with NCL in A549 cells. (A) Immunofluorescence of Flag-tagged NS1 (Green) or NCL (Red) in NS1 transfected A549 cells. (B) Cell lysates of mock-transfected or NS1-transfected A549 cells were subjected to immunoprecipitation using anti-Flag antibody and immunoblotted with anti-NCL or anti-Flag antibodies. For each, 5% cell lysate was served as input.

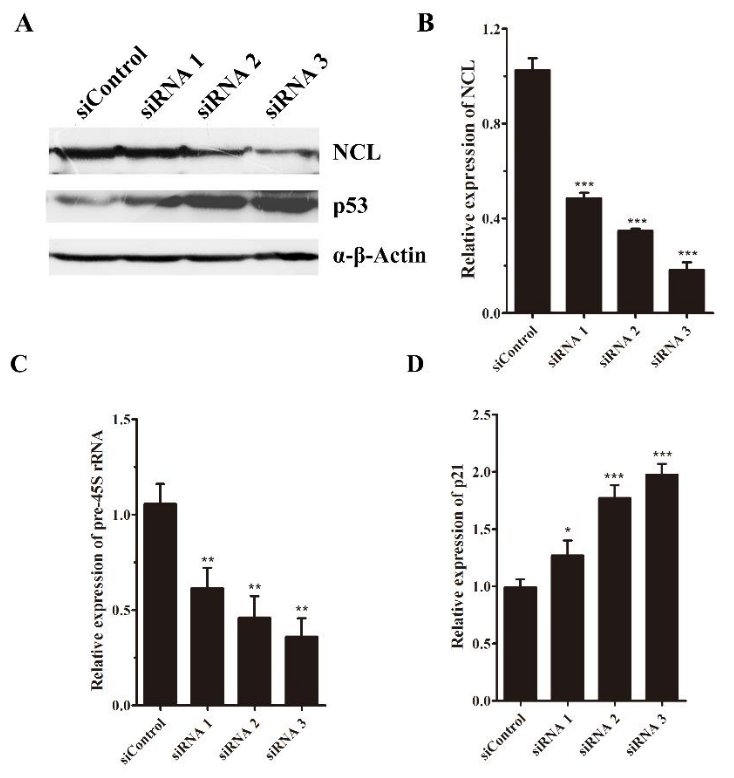

Figure S3. Knockdown of NCL induces nuclolar stress in A549 cells. A549 cells treated with NCL-targeting siRNAs for 72 h and subjected to western blot analysis and RT-qPCR. (A) NCL and p53 expression level of siRNA-treated A549 cells. mRNA levels of NCL (B), pre-45S rRNA (C) and p21 (D) in siRNA-treated A549 cells. A scrambled sequence was served as control. **p* < 0.05, ***p* < 0.01, *** *p* < 0.001 (*t-test*). siRNA Oligonucleotides were listed in Table S5.

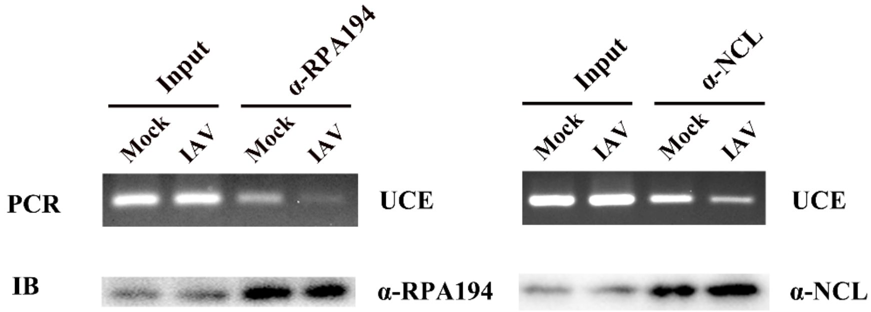

Figure S4. ChIP of RPA194 and NCL with UCE in mock-infected or IAV-infected A549 cells.

Chromatin immunoprecipitation of RPA194 (left panels) or NCL (right panels) with UCE in mock-infected or IAV-infected A549 cells. Immunoprecipitated DNA was analyzed by PCR using the primers shown in Table S4.

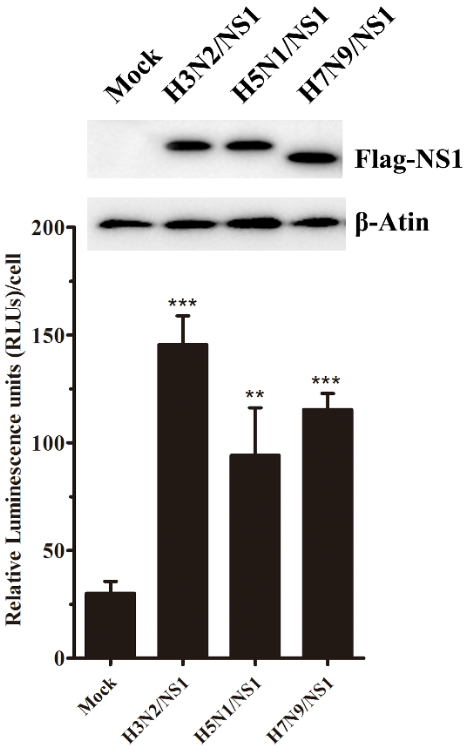

Figure S5. Caspase 3/7 activity assay of H3N2/NS1, H5N1/NS1 or H7N9/NS1-transfected A549 cells. Cells were harvested at 36 h post-transfection and assayed for caspase 3/7 activity. Each treatment was performed in triplicate. $^{**}p < 0.01$, $^{***}p < 0.001$ (*t-test*).

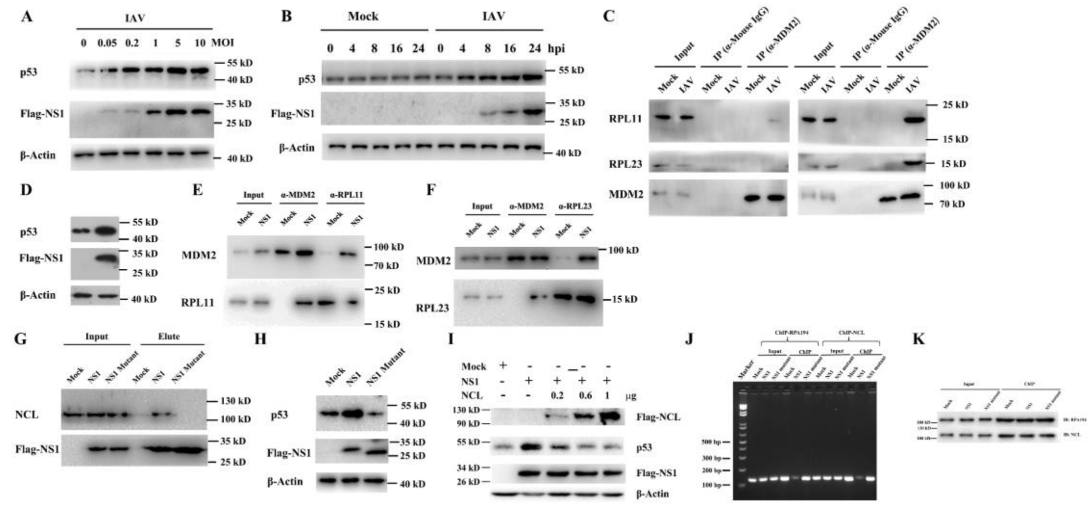

Figure S6. Full-length blots of the immunoblotting detection and full-length gel of ChIP. (A), (B) and (C): Full size blots of immunoblotting detection shown in Figure 1A, 1D and 1G. (D): Full size blots of immunoblotting detection shown in Figure 2A. (E) and (F): Full size blots of immunoblotting detection shown in Figure 2D. (G) and (H): Full size blots of immunoblotting detection shown in Figure 3B and Figure 3C. (I): Full size blots of immunoblotting detection shown in Figure 4A. (J): Full-length gel of Figure 5A upper panel. (K): Full size blots of Figure 5A lower panel.

Supplemental Materials and Methods

siRNA interference

RNA interference was performed by using synthetic siRNA duplexes. siRNA oligonucleotides targeting nucleolin were designed and synthesized by Genepharma (Shanghai, China). All siRNA oligonucleotide sequences are listed in Table S5. Cells were transfected with 150 ng siRNA/well in 6-well plates for 72 h, using Lipofectamine 2000 (Invitrogen) based on the manufacturer's instructions.

Apoptosis Assays

A549 cells were seeded in 96-well tissue culture plates at a density of 1×10^4 cells per well in 100 μl of medium and allowed to adhere for 36 h. Cells were transfected with 0.2 μg DNA plasmid in triplicate. Caspase 3/7 activity was determined 36 h post-transfection using the Caspase-Glo 3/7 assay (Promega) following the manufacturer's manual. Luminescence activities (relative caspase 3 and 7 activities) were measured as relative light units (RLUs) with GloMax Multi+ Luminometer (Promega).

Supplemental Tables

Table S1. Primers for qPCR

Gene	Sequences(5'-3')
pre-45S rRNA	F: GCCTTCTCTAGCGATCTGAGAG
	R: CCATAACGGAGGCAGAGACA
NCL	F: ACCCAGGGGATCACCTAATG
	R: CCTTGGAGGACCCAGTTTC
P21	F: AAGACCATGTGGACCTGTCACTGT
	R: GAAGATCAGCCGGCGTTTG
β-Actin	F: ATGGGTCAGAAGGATTCCTATGT
	R: GGTCATCTTCTCGCGGTT

Table S2. Primers for cloning

Gene	Sequences(5'-3')
NS1	F: TATGGATCCATGGATTCCAACACTGTG
	R: TACGGATCCTCAAACGTTTTGACCTAGC
NS1 Mutant	F: AATGGATCCATGGATTCCAACACTGTG
	R:CCGCTCGAGTCATTTTGGAGTAAGTGG
NCL	F:CCGGAATTCATGGTGAAGCTCGCGAAGGCAG
	R:CCGCTCGAGCTATTCAAACTTCGTCTTCTTTC

*Sequences of restrict cutting sites are underlined for emphasizing

Table S3. Primers for ChIP

Primer Name	Sequences(5'-3')
UCE	F: CGTGTGTCCTTGGGTTGACC
	R: CGCGTCACCGACCACGCC

Table S4. Primers used in DNA methylation analysis

Primer Name	Sequences(5'-3')
mUCE	F: TGTGTTTTGGGGTTGATTAGAG
	R: AAAACCCAACCTCTCCAA

Table S5. siRNA Oligonucleotides

siRNA Name	Sequences(5'-3')
siControl	sense: UUCUCCGAACGUGUCACGUTT<br>antisense: ACGUGACACGUUCGGAGAATT
siRNA 1	sense: CCUGCCAAGAAGACAGUUATT<br>antisense: UAACUGUCUUCUUGGCAGGTT
siRNA 2	sense: GGCGAUCUAUUUCCCUGUATT<br>antisense: UACAGGGAAAUAGAUCGCCTT
siRNA 3	sense: CGGCUUUCAAUCUCUUUGUTT<br>antisense: ACAAAGAGAUUGAAAGCCGTT
